# EV-D68 exploits clathrin-mediated endocytosis and compensatory macropinocytosis for cellular entry

**DOI:** 10.1128/jvi.00358-26

**Published:** 2026-04-27

**Authors:** Chia-Yi Lin, Wen-Fang Tang, Kuang-Jing Huang, Li-Ting Wang, Jie-Yun Cai, Cheng-Hsun Chiu, Jim-Tong Horng

**Affiliations:** 1 Graduate Institute of Biomedical Sciences, College of Medicine, Chang Gung University, Taoyuan, Taiwan; 2 Research Center for Emerging Viral Infections, College of Medicine, Chang Gung University 56081, Taoyuan, Taiwan; 3 Department of Biochemistry and Molecular Biology, College of Medicine, Chang Gung University 56081, Taoyuan, Taiwan; 4 Molecular Infectious Disease Research Center, Chang Gung Memorial Hospital, Chang Gung University College of Medicine 71589, Taoyuan, Taiwan; 5 Center for Drug Research and Development, Chang Gung University of Science and Technology 63113, Taoyuan, Taiwan; Loyola University Chicago - Health Sciences Campus, Maywood, Illinois, USA

**Keywords:** enterovirus D68, entry, clathrin-mediated endocytosis, macropinocytosis, membrane-associated virus

## Abstract

Despite growing health concerns about enterovirus D68 (EV-D68), its cellular entry mechanisms remain incompletely understood. In this study, we investigated the endocytic pathways exploited by the sialic acid-dependent EV-D68 TW-02795-2014 strain using pharmacological inhibitors, siRNA-mediated knockdown, dominant-negative constructs, and electron microscopic analysis. Chemical inhibition of clathrin-mediated endocytosis (CME), macropinocytosis, endosomal acidification, and other endocytic pathways reduced viral entry and protein expression, suggesting the involvement of multiple routes in viral entry. Co-localization of EV-D68 with clathrin and the effects of CME inhibitors supported CME as a major entry route. However, siRNA knockdown of CME components in RD cells inhibited the cellular uptake of transferrin, yet had no significant impact on EV-D68 infection. This was also seen in A549 cells. Notably, CME disruption by clathrin knockdown led to Rac1 upregulation and increased macropinocytosis, which facilitated EV-D68 entry. Transmission electron microscopy revealed membrane-associated EV-D68 particles organized in clusters underneath the cell surface during infection, potentially undergoing bulk internalization. In contrast, purified virions lacking membranous material strictly required CME for successful infection. These findings reveal a dual entry strategy: single (membrane-free) EV-D68 particles utilize CME, while membrane-associated virions access host cells via macropinocytosis, particularly under conditions of CME inhibition. This study highlights the complexity of EV-D68 endocytosis and underscores the importance of compensatory mechanisms in viral uptake.

**IMPORTANCE:**

Understanding how EV-D68 enters host cells is crucial for developing antiviral strategies. This study uncovers a dual-entry mechanism used by a sialic acid-dependent EV-D68 strain: clathrin-mediated endocytosis (CME) for single virus particles, and macropinocytosis as an alternative route for membrane-associated particles or under conditions where CME is impaired. Notably, disruption of CME induces a compensatory upregulation of macropinocytosis, mediated by increased Rac1. These findings challenge prior assumptions of a singular viral entry pathway and emphasize the need to consider endocytic plasticity when designing antiviral interventions targeting EV-D68 or similar viruses.

## INTRODUCTION

Enterovirus D68 (EV-D68) belongs to the genus Enterovirus, species Enterovirus D in the family Picornaviridae (1, 2). Despite its initial identification in 1962 from four hospitalized children in the United States with pneumonia (3), subsequent reported cases have been limited, primarily displaying respiratory symptoms (4–6). Since 2014, several EV-D68 infections have been associated with severe neurological complications like acute flaccid myelitis (AFM) (7, 8) and even paralysis, suggesting a potential link between EV-D68 and poliovirus (Enterovirus C). This underscores the importance of managing EV-D68 infections and curbing its spread. However, the supported treatments were the usual way of clinical care for EV-D68-infected patients. Currently, there are no approved vaccines or antiviral agents specifically targeting EV-D68. A thorough understanding of the viral infection mechanisms is essential for the development of effective preventive and therapeutic interventions.

Endocytosis can be categorized into phagocytosis and pinocytosis (9). The latter encompasses various pathways characterized by distinct morphologies, molecular components, and specific machinery, including clathrin-mediated endocytosis (CME) (10–13), caveolae-mediated endocytosis (14, 15), and less well-characterized routes such as macropinocytosis (16, 17). Previous studies have identified multiple glycans and host factors involved in EV-D68 attachment and entry. EV-D68 can bind several forms of sialic acid as critical receptors—including α−2,3 sialic acid, α−2,6 sialic acid, similar to the influenza A virus (IAV), and it can also attach to α−2,8 sialic acid-containing glycolipids (18–20). In addition to sialylated glycans, sulfated glycosaminoglycans (sGAGs), the glycoprotein ICAM-5, and MFSD6 have been reported to support EV-D68 binding and internalization, particularly in sialic acid-independent strains (21–24). Virus entry pathways are closely linked to their receptors (25, 26), indicating that EV-D68 may utilize similar endocytic pathways as IAV, such as CME and macropinocytosis (27–29).

While previous studies have hinted at the involvement of CME in EV-D68 entry through chemical inhibitor-treated evidence (21, 30), the precise entry mechanism of EV-D68 has not been extensively described, unlike the comprehensive investigations of other viruses. Therefore, further research is necessary to determine whether EV-D68 utilizes the same host endocytic pathways as the IAV or involves novel and undiscovered endocytosis mechanisms that facilitate its entry. In this study, our findings suggest that several endocytosis inhibitors effectively combat EV-D68, highlighting the potential use of multiple endocytic pathways for virus internalization. These results were corroborated by fluorescence confocal microscopy and transmission electron microscopy (TEM) images. The ineffectiveness of CME component modulation in altering infection, combined with the virus’s ability to bind diverse sialylated proteins, suggests a flexible entry strategy involving both CME and macropinocytosis, underscoring the complexity of EV-D68 entry and highlighting the need for further research to fully elucidate its endocytic mechanisms.

## RESULTS

### Identifying the sialic acid dependency of EV-D68 TW-02795-2014

**Fig 1:**
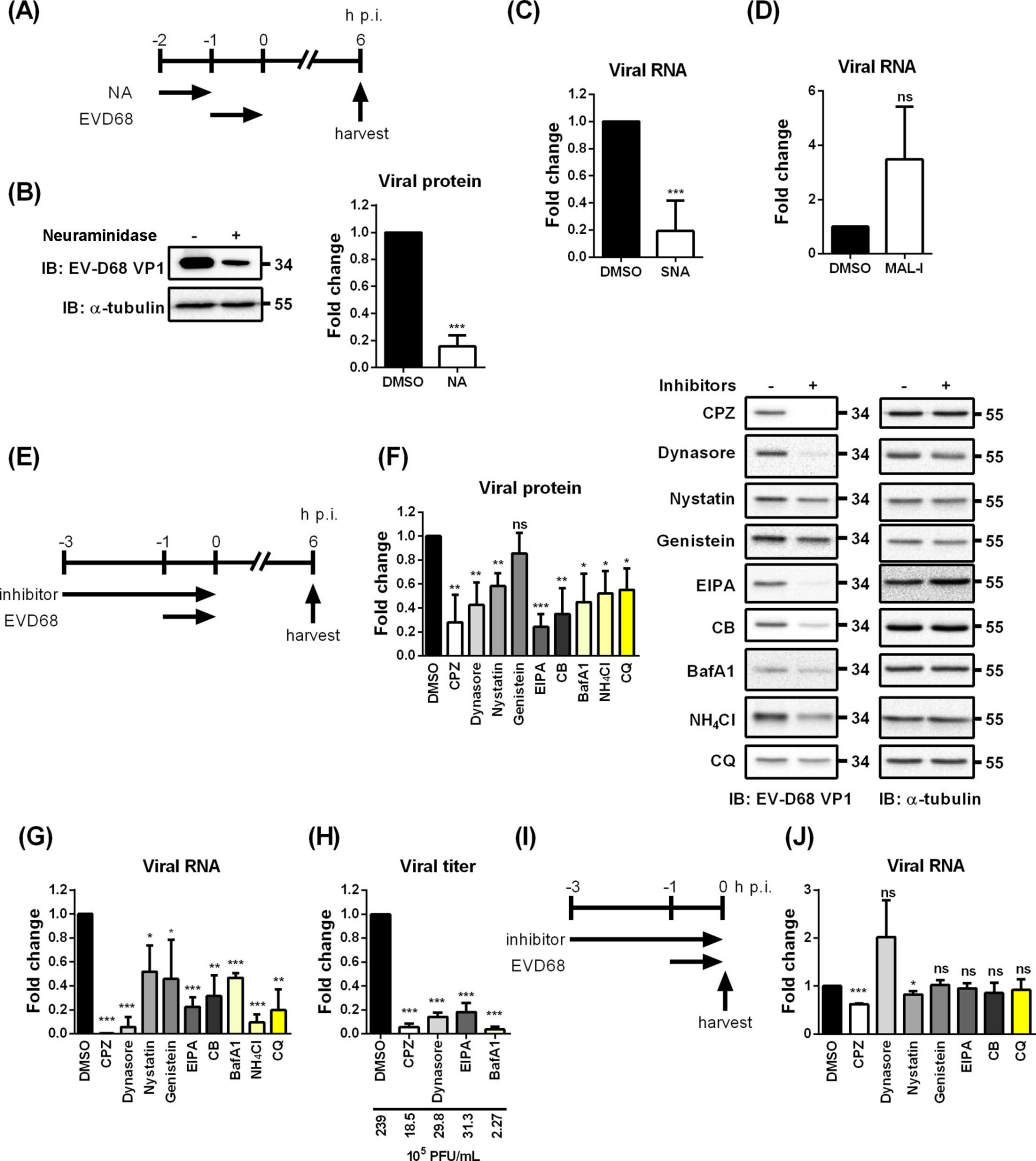
EV-D68 TW-02795-2014 infection is dependent on sialic acid and inhibited by endocytic inhibitors. (**A**) Schematic illustration of neuraminidase treatment protocol. (**B**) RD cells were pre-treated with 50 mU of neuraminidase for 1 h and subsequently infected with EV-D68 at an MOI of 10. The cells were harvested at 6 h p.i., and viral protein expression was analyzed by immunoblotting with specific antibodies against VP1. Quantification of signal intensity is shown in the right panel. (**C–D**) RD cells were pre-treated with SNA or MAL-I and infected with EV-D68 at an MOI of 1. Viral RNA levels were harvested at 6 h p.i. for qRT-PCR analysis. (**E**) Schematic illustration of inhibitor treatment. The cells were pre-treated with chemical inhibitors in DMEM for 2 h (−3 to −1 h p.i.) at 37°C. EV-D68 (MOI = 1) was incubated with cells in inhibitor-containing DMEM for 1 h at 33°C. The cells were incubated in E2 for 6 h at 33°C. The viral protein, viral RNA, and viral production were analyzed with immunoblotting (**F**), qRT-PCR (**G**), and plaque assay (**H**), respectively. Immunoblotting images represent three independent replicates. (**I**) RD cells were pre-treated with selected chemical inhibitors in DMEM for 2 h at 37°C. EV-D68 was incubated with cells in inhibitor-containing DMEM at an MOI of 100 for 1 h at 33°C. The virus and inhibitor were removed by PBS washing. (**J**) The inhibition of viral RNA was analyzed with qRT-PCR. Data are presented as means ± SD of three replicates; comparisons were carried out using the Student’s *t*-test (*, *P* < 0.05; **, *P* < 0.01; ***, *P* < 0.001; and ns, no significance).

In this study, we primarily used the EV-D68 isolate TW-02795-2014, which belongs to clade B3 and was isolated from a female patient who developed severe symptoms and fatal complications (31). Pretreatment of RD cells with neuraminidase to remove sialic acids prior to EV-D68 adsorption markedly inhibited viral infection and diminished viral protein expression at 6 h post-infection (h p.i., Fig. 1A and B). In addition, competition with Sambucus nigra agglutinin (SNA), a lectin that specifically binds α−2,6-linked sialic acid, markedly reduced viral attachment, whereas competition with Maackia amurensis lectin I (MAL-I), which binds α2,3-linked sialic acids, did not inhibit infection (Fig. 1C and D). Together, these findings indicate that EV-D68 is highly dependent on α−2,6 sialic acid for host cell entry.

### Endocytosis inhibitors treatment reveals the multiple entry routes for EV-D68 infection

In order to identify potential endocytic pathways that were involved in EV-D68 infection, we first used nine inhibitors to target multiple steps in known endocytic pathways dependent or independent of clathrin (Fig. 1E through H). Chemical inhibition of various stages of CME—including coated pit formation by chlorpromazine (CPZ) and cytochalasin B (CB), membrane scission by dynasore, coat protein uncoating by CPZ, and endosomal acidification by bafilomycin A1 (BafA1), ammonium chloride (NH_4_Cl), and chloroquine (CQ). Inhibitors targeting clathrin-independent pathways, such as caveolin-mediated (genistein and nystatin), cholesterol-dependent endocytosis (nystatin), and macropinocytosis (EIPA) were also included in the assay. The working concentrations of the inhibitor were based on values reported in the literature and were confirmed to result in less than 20% cytotoxicity in our experiments (data not shown) (25, 26, 32). To investigate the inhibitory effects on early stages of EV-D68 infection, RD cells underwent a 3-h treatment with the inhibitors before and during viral adsorption. After incubation, both the virus and inhibitors were removed, intracellular RNA, proteins, and progeny virus were collected for analysis at 6 h p.i. (Fig. 1E). All inhibitors significantly suppressed viral protein synthesis, viral genomic RNA accumulation, and virus titer relative to the solvent control (Fig. 1F through H). These findings are in line with previous studies on EV-D68, which largely focused on receptor interactions and commonly included pharmacological inhibition data to support their conclusions regarding entry routes (21). However, our results further indicate a broader range of endocytic strategies employed by the sialic acid-dependent EV-D68 TW-02795-2014.

To further confirm that the inhibition of EV-D68 occurred at an early stage, we assessed the viral RNA levels after a 1-h infection with a high viral load (MOI = 100) (Fig. 1I). Treatment with CPZ resulted in a greater reduction in viral RNA compared to other inhibitors, indicating that CME plays a role in EV-D68 attachment and entry. However, treatment with dynasore yielded opposite results (Fig. 1J). The increased viral RNA might result from virions trapped in the “pit” of the cell surface, unable to complete scission due to the lack of functional dynamin. Based on the inhibitor treatment results, we suspect that CME serves as the primary entry route for EV-D68, with macropinocytosis and cholesterol-dependent endocytosis acting as alternative and minor pathways.

### Co-localization of EV-D68 capsid protein VP1 and clathrin at early infection stage

**Fig 2:**
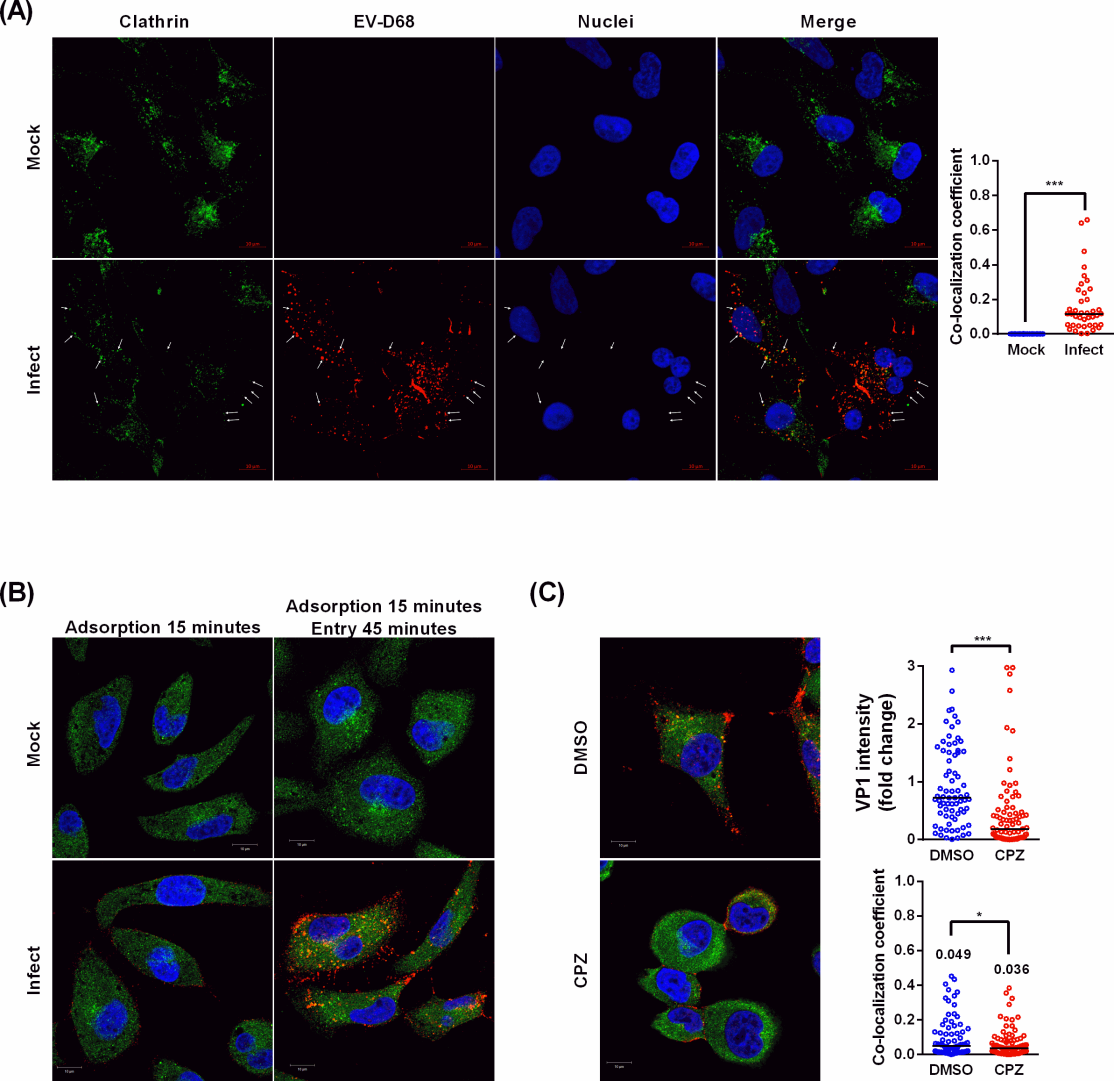
Clathrin and EV-D68 VP1 co-localization during early stages of infection and disruption by chemical inhibitor. Subcellular localization of EV-D68 VP1 (red) and clathrin (green), as well as their co-localization, was analyzed by immunofluorescence confocal microscopy using antibodies specific to clathrin heavy chain and VP1. (**A**) EV-D68 was incubated with RD cells in DMEM at an MOI of 200 for 1 h at 33°C. More than 30 cells per group were quantified using Zeiss Zen software. (**B**) EV-D68 strain was incubated with RD cells in DMEM at an MOI of 100 for 15 min at 4°C. The cells were then transferred to 33°C to facilitate viral entry. (**C**) RD cells were treated with CPZ after virus adsorption and incubated with EV-D68 at 33°C for 45 min. More than 30 cells per group were quantified using Zeiss Zen software, with median values indicated. Data are presented as the median for each group. Statistical comparisons were performed using the Mann–Whitney *U*-test (*, *P* < 0.05; **, *P* < 0.01; and ***, *P* < 0.001).

To further explore the involvement of CME in EV-D68 infection, we conducted an immunofluorescence confocal microscopy to assess the subcellular distribution of clathrin and the viral capsid protein VP1 during the early stage of infection. Following a 1-h infection at high viral load under physiological temperature (33°C) conditions, EV-D68 (red) partially co-localized with clathrin (green), as indicated by the arrows (Fig. 2A). Since endocytosis occurs rapidly and clathrin triskelion uncoats during intracellular transport, the virus capsid protein is expected to transiently co-localize with it. RD cells were infected with EV-D68 at 4°C and then shifted to 33°C to synchronize the entry events (Fig. 2B). The VP1 was not observed when incubated at 4°C but became more intense and migrated from the cell surface to the intracellular space, with partial co-localization with clathrin. This phenomenon was not observed in CPZ-treated cells, indicating that CPZ treatment markedly restricts the entry of EV-D68 (Fig. 2C), implying that CME is one of the entry routes for EV-D68 internalization.

### Specifically targeting CME component molecules produces opposing effects on EV-D68 infection

**Fig 3:**
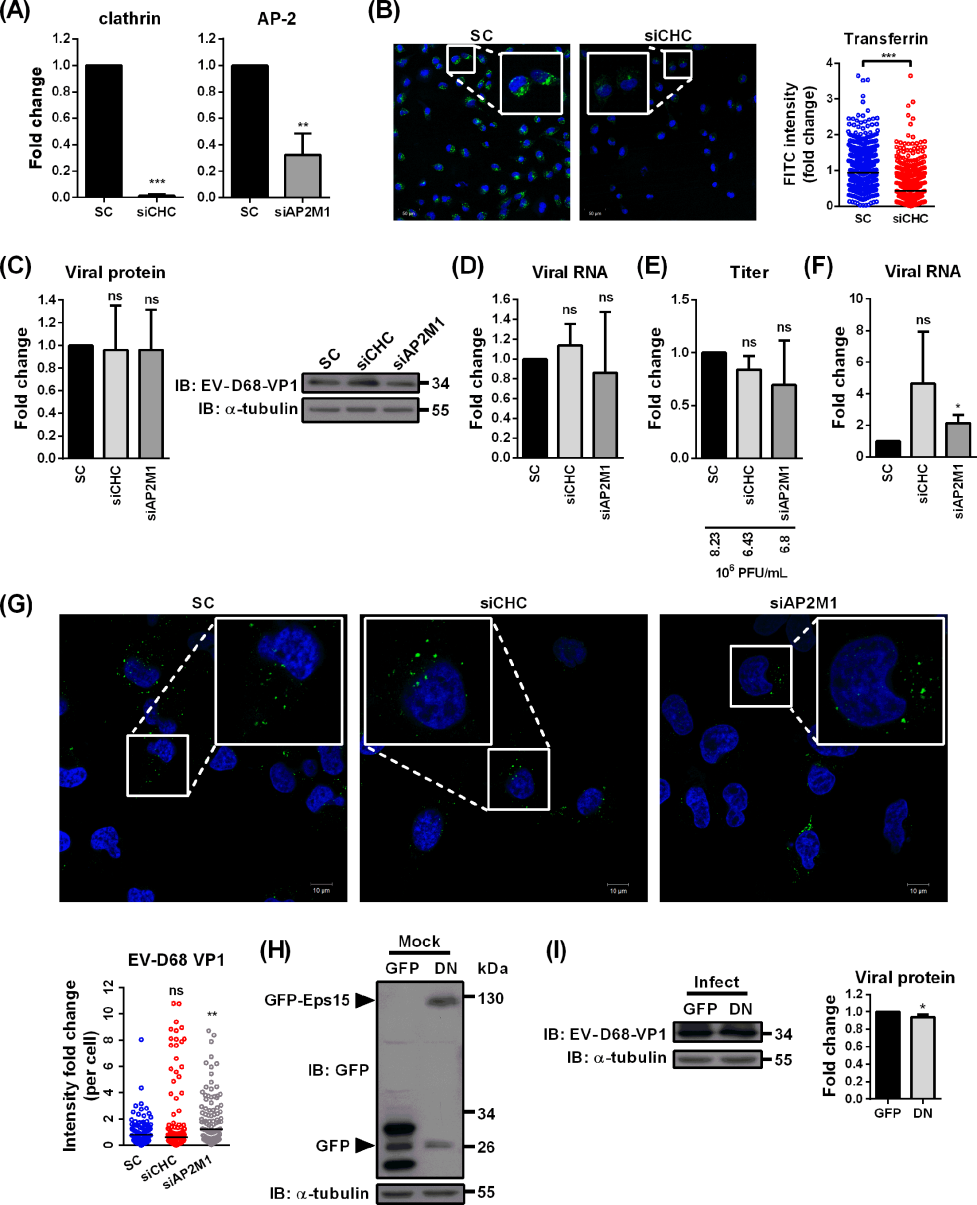
Disruption of clathrin-mediated endocytosis through siRNA knockdown or dominant-negative Eps15 overexpression did not affect EV-D68 infection in RD cells. (**A**) The siRNA of clathrin and AP2 was transfected in RD cells for 3 days, and the knockdown efficiency was measured by qRT-PCR. (**B**) Transferrin-FITC (green) and Hoechst (blue) were added to transfected cells and incubated at 33°C for 1 h, followed by fixation with 4% PFA. The FITC intensity in each cell was quantified using Investigator software, and statistical analysis was performed using the Mann–Whitney *U*-test. (**C–E**) EV-D68 was incubated with cells in DMEM at an MOI of 1 for 1 h at 33°C. Inhibition of viral replication in the context of expression of viral protein, viral RNA, and viral titers in RD cells transfected with siCHC and siAP2M1. The viral protein, viral RNA, and viral production were analyzed with immunoblotting (**C**), qRT-PCR (**D**), and plaque assay (**E**), respectively. Immunoblotting images represent three independent replicates. Mean raw virus titers are indicated. (**F**) siCHC or siAP2M1 was transfected in RD cells for 3 days. Cells were incubated with EV-D68 (MOI 1) for 1 h at 4°C, after which the inoculum was removed, and the cells were incubated in E2 at 33°C for 1 h. The cells were treated with trypsin prior to harvesting for qRT-PCR analysis. (**G**) The EV-D68 was incubated with transfected cells in DMEM at an MOI of 100 for 15 min at 4°C and transferred to 33°C to facilitate entry for 45 min to allow viral entry. EV-D68 VP1 (green) and nuclei (blue) were stained using an anti-EV-D68 VP1 antibody and Hoechst, respectively. Subcellular VP1 immunofoci were quantified using Zen software. Results are representative of four independent replicates. (**H and I**) Plasmids of dominant-negative Eps15 or its EGFP-C2 vector control were transfected into RD cells for 16 h. The cells were then infected with EV-D68 at an MOI of 1 and harvested at 6 h p.i. The expression levels of GFP-dominant-negative Eps15 (**H**) and viral protein (**I**) were detected by immunoblotting. Immunoblotting images were representative of three independent replicates. Data are presented as means ± SD of three replicates; comparisons were carried out using the Student’s *t*-test (*, *P* < 0.05; **, *P* < 0.01; ***, *P* < 0.001; and ns, no significance).

**Fig 4:**
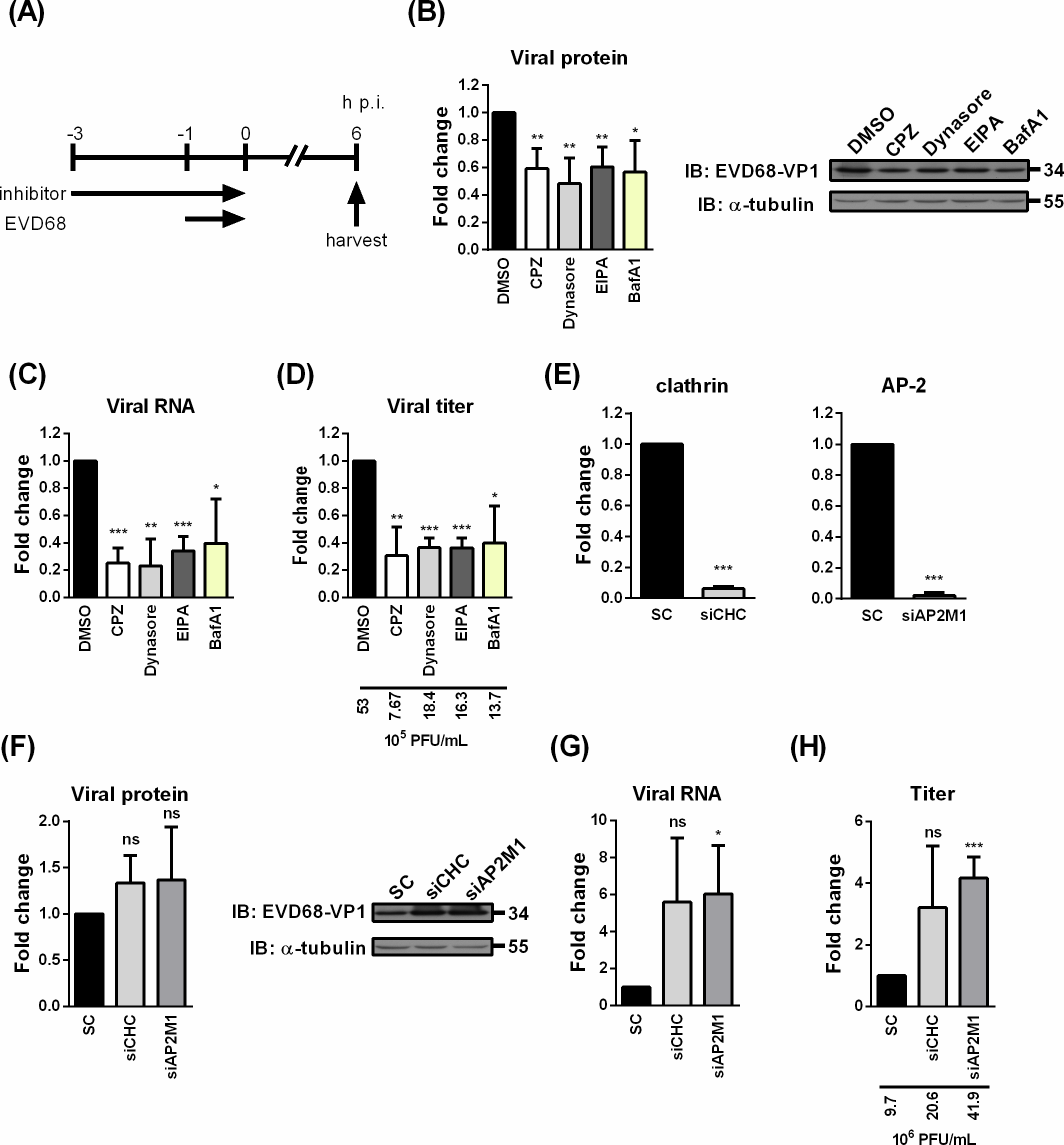
Inhibitory effects of chemical inhibitors and CME component modulation on EV-D68 infection in A549 cells. (**A**) Schematic illustration of inhibitor treatment. A549 cells were treated with chemical inhibitors using the same conditions as applied to RD cells in Fig. 1D. The viral protein, viral RNA, and viral production were analyzed with immunoblotting (**B**), qRT-PCR (**C**), and plaque assay (**D**), respectively. Immunoblotting images represent three independent replicates. (**E–H**) Knockdown of clathrin and AP-2 in EV-D68 infection in A549 cells. The siRNA of clathrin and AP2 was transfected in A549 cells for 3 days, and knockdown efficiency was measured by qRT-PCR (**E**). The cells were infected with EV-D68 at an MOI of 1 in DMEM for 1 h at 33°C, followed by a 6-h incubation in E2. Viral protein, RNA, and production were assessed by immunoblotting (**F**), qRT-PCR (**G**), and plaque assay (**H**), respectively. Immunoblotting images represent three independent replicates. Mean raw virus titers are indicated. Data are presented as means ± SD of three replicates; comparisons were carried out using the Student’s *t*-test (*, *P* < 0.05; **, *P* < 0.01; ***, *P* < 0.001; and ns, no significance).

We further investigated the significance of CME in EV-D68 infection by employing overexpression of dominant-negative Eps15 and downregulation of clathrin or adaptor-related protein complex 2 (AP-2) (Fig. 3). Specifically, targeting Eps15 and AP-2 reduces clathrin-coated pit formation, thereby impairing cargo internalization through CME, including transferrin uptake (33). Transfection with specific siRNAs led to a marked decrease in clathrin and AP-2 mRNA expression in RD cells, indicating effective gene silencing in the treated cells (Fig. 3A). The corresponding decrease in transferrin-FITC uptake further indicates that knockdown of clathrin effectively disrupts CME (Fig. 3B). However, despite efficient disruption of CME, silencing endogenous clathrin or AP-2 did not significantly affect EV-D68 infection, as evidenced by unchanged levels of viral protein expression, RNA accumulation, and progeny virus production (Fig. 3C through E). Consistently, neither viral RNA levels nor VP1 signal intensity at the early stage of infection was reduced in clathrin- or AP-2-depleted cells (Fig. 3F and G). Similarly, cells expressing dominant-negative Eps15 showed only a slight reduction in expression of VP1 compared to cells expressing GFP vector only (Fig. 3H and I). These results show that disrupting CME, whether by clathrin or AP-2 knockdown or dominant-negative Eps15 overexpression, does not affect EV-D68 infection. This contradicts previous published findings observed in other enteroviruses, such as EV-A71 (25, 32, 34). To rule out the possibility of cell-type specificity, we repeated both the inhibitor treatments and gene silencing experiments in A549 cells (Fig. 4). While chemical inhibitor treatments significantly suppressed EV-D68 infection (Fig. 4A through D), knockdown of clathrin and AP-2 did not reduce viral protein expression, viral RNA accumulation, and progeny virus production (Fig. 4E through H), consistent with our observations in RD cells. These inconsistent findings raise the question of whether CME is truly the primary endocytic route for EV-D68 infection or if alternative pathways are involved.

### The diverse EV-D68 release populations and potential alternative entry pathways

**Fig 5:**
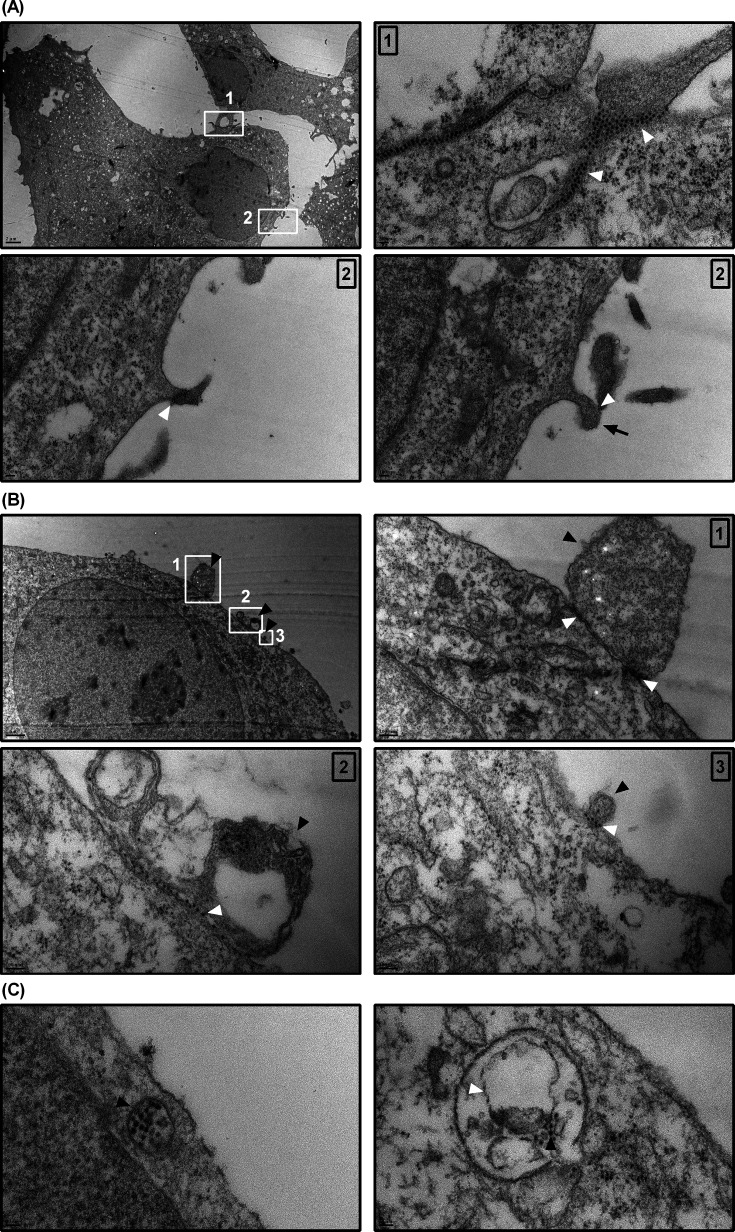
Transmission electron microscopy images of infected cell surface and large membrane compartment-associated EV-D68. Canonically purified and concentrated EV-D68 using 100 kDa MWCO Amicon ultra centrifugal filters was incubated with RD cells in DMEM at an MOI of 7,000 for 1 h at 33°C. (**A**) Clustering of EV-D68 particles (Area 1, white arrowheads) and membrane protrusion (Area 2, black arrow). The insets display the images at higher magnification. (**B**) EV-D68 was observed in the junction between the plasma membrane and the large membrane structures (black arrowheads in Areas 1–3) and arrays of viral particles were observed under higher magnification in the insets (white arrowheads in 1 and 2). (**C**) Internalized EV-D68 particles and associated membrane structures enclosed within intracellular vesicles.

To further investigate our findings, we utilized transmission electron microscopy (TEM) to visualize early events in the viral infection process at high resolution. We used higher infection doses than those applied in IFA, enabling easier detection of viral particles during the early stages of infection. Interestingly, TEM revealed that RD cells displayed numerous EV-D68 particles aligned beneath the cell surface through membrane invagination, a pattern distinct from classical coated pit formation (Fig. 5A-1). These clustered membrane-invagination events involving multiple viral particles overshadowed the observations of single-particle uptake. We also observed structures resembling membrane protrusions associated with EV-D68 on the cell surface (Fig. 5A-2). Interestingly, virus particles were localized between the plasma membrane and various membrane compartments (Fig. 5B). At higher magnification, ordered arrays of viral particles were clearly observed (panels 2 and 3 of Fig. 5B). These virus particles, together with their associated membrane compartments, were subsequently internalized (Fig. 5C). TEM images revealed two key observations: clathrin-independent endocytosis and membrane compartment-associated viruses. Notably, EV-D68 particles were not observed within CME-related structures, including clathrin-coated pits or clathrin-coated vesicles, in the TEM images analyzed. Based on recent research on EV-D68 and extracellular vesicles (35, 36), we suspect that these large membrane compartments seen in Fig. 5B are associated with virus particles upon release. The large membrane compartments could pose an obstacle, contributing to the difficulty in observing the entry of single virus particles. Furthermore, the membranous structures associated with virus particles may facilitate membrane-mediated endocytosis rather than direct virus particle-receptor mediated entry (37, 38), contributing to the complexity of viral entry mechanisms. Combined with results from chemical inhibitor treatments, this provides insight into the possibility that membrane-associated EV-D68 may be internalized through clathrin-independent endocytosis.

### Virus-associated membrane compartments and compensatory macropinocytosis influence EV-D68 entry routes

Because CME is a pathway for the uptake of extracellular materials and the regulation of cell-surface protein turnover, downregulation of clathrin is expected to disrupt endocytic homeostasis. Previous studies have suggested that impairment of CME can trigger compensatory activation of alternative endocytic pathways to sustain cellular uptake of essential extracellular components. This raises the possibility that EV-D68 exploits alternative entry routes that become enhanced upon CME disruption. Consistent with this notion, EV-D68 was sensitive to inhibitors targeting multiple endocytic pathways observed in Fig. 1, supporting the involvement of alternative uptake mechanisms. Based on reports describing the dual usage of endocytic pathways by IAV, we hypothesized that EV-D68 utilizes both CME and macropinocytosis for infection, with macropinocytosis being upregulated under CME-deficient conditions.

**Fig 6:**
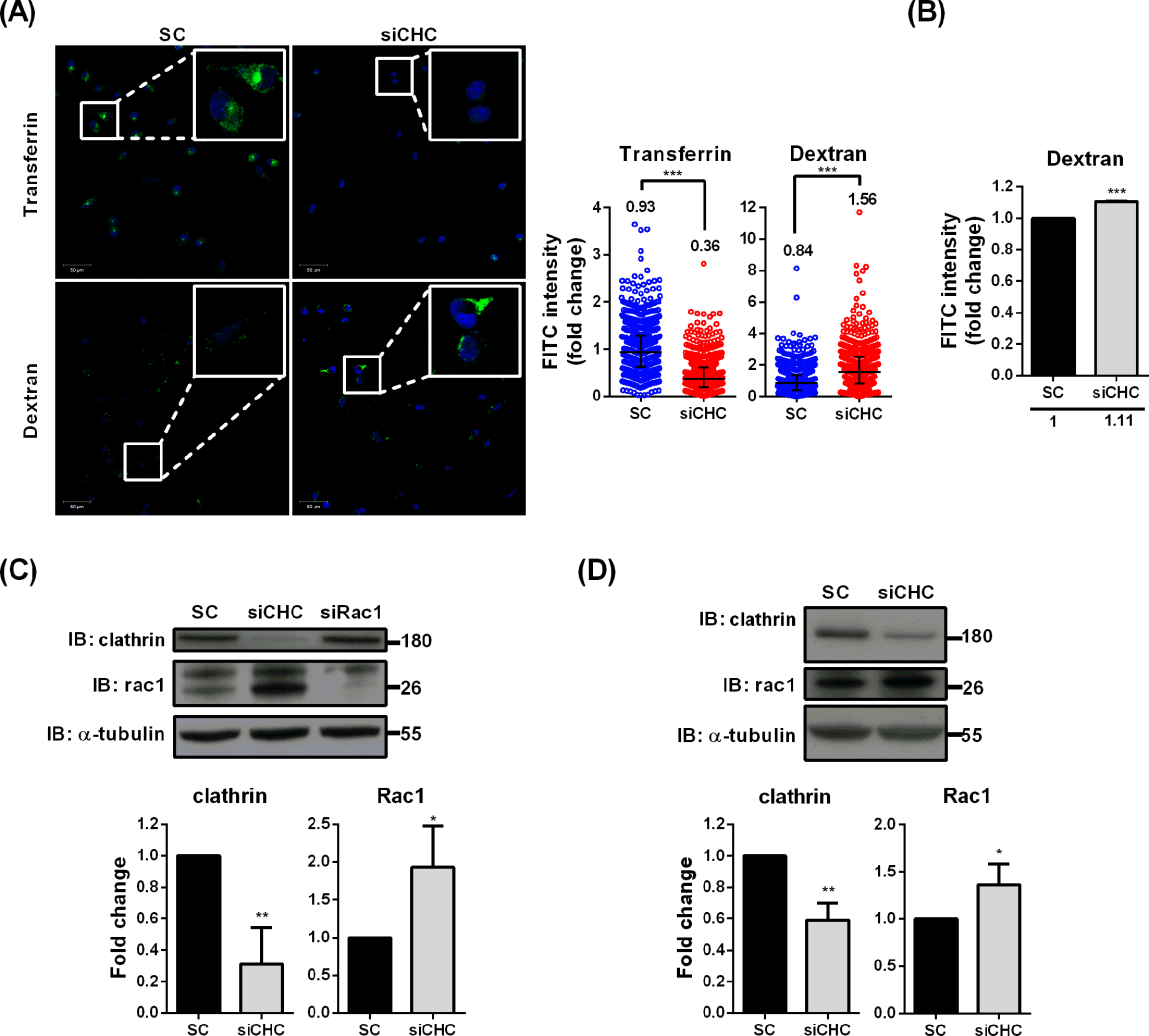
Downregulation of clathrin leads to Rac1 upregulation and compensatory activation of macropinocytosis. (**A**) RD cells were transfected with siRNA for 3 days and then treated with transferrin-FITC or dextran-FITC for 1 h at 33°C. The nucleus was stained with Hoechst, and FITC intensities were quantified using Investigator software from four independent replicates; representative images from one replicate are shown, with median values indicated. (**B**) Flow cytometric analysis of dextran-FITC uptake in control (SC) and clathrin-depleted (siCHC) cells from four independent experiments, with mean values indicated. (**C and D**) Rac1 expression level was increased in siCHC-transfected RD and A549 cells. Clathrin and Rac1 expression levels in siRNA-transfected RD (**C**) and A549 (**D**) were analyzed by immunoblotting. The images represent the three independent replicates. Data are presented as means ± SD of three replicates; comparisons were carried out using the Student’s *t*-test and Mann-Whitney *U*-test (*, *P* < 0.05; **, *P* < 0.01; and ***, *P* < 0.001).

To determine whether macropinocytosis is upregulated as a compensatory response in clathrin-deficient cells, we assessed endocytic activity using fluorescent uptake assays. Control and clathrin knockdown cells were incubated with transferrin–FITC or dextran–FITC to monitor CME and macropinocytosis, respectively. Because dextran–FITC exhibited a scattered distribution across multiple focal planes, whereas transferrin–FITC was more centralized, dextran uptake was quantified using two approaches. Following the method described by Kuriyama et al. (39), fluorescence microscopy (Fig. 6A) and flow cytometry (Fig. 6B) produced similar trends. To minimize subjective bias from image focusing, subsequent experiments quantified dextran–FITC uptake exclusively by flow cytometry. As expected, transferrin–FITC uptake was markedly reduced in clathrin knockdown cells, confirming effective inhibition of CME. In contrast, dextran–FITC uptake was significantly increased (Fig. 6A and B), indicating elevated macropinocytosis activity. These findings suggest that prolonged CME disruption triggers compensatory activation of macropinocytosis to maintain overall endocytic capacity.

To further investigate the mechanism underlying increased macropinocytosis, we analyzed the expression of Rac1, a key regulator of actin polymerization and membrane ruffle formation during macropinocytosis (40). Clathrin knockdown resulted in increased Rac1 expression in both RD (Fig. 6C) and A549 cells (Fig. 6D), indicating that this response is not cell-type specific. Together, these findings suggest that clathrin depletion induces a general compensatory response characterized by Rac1 upregulation and enhanced macropinocytosis in response to CME dysfunction.

**Fig 7:**
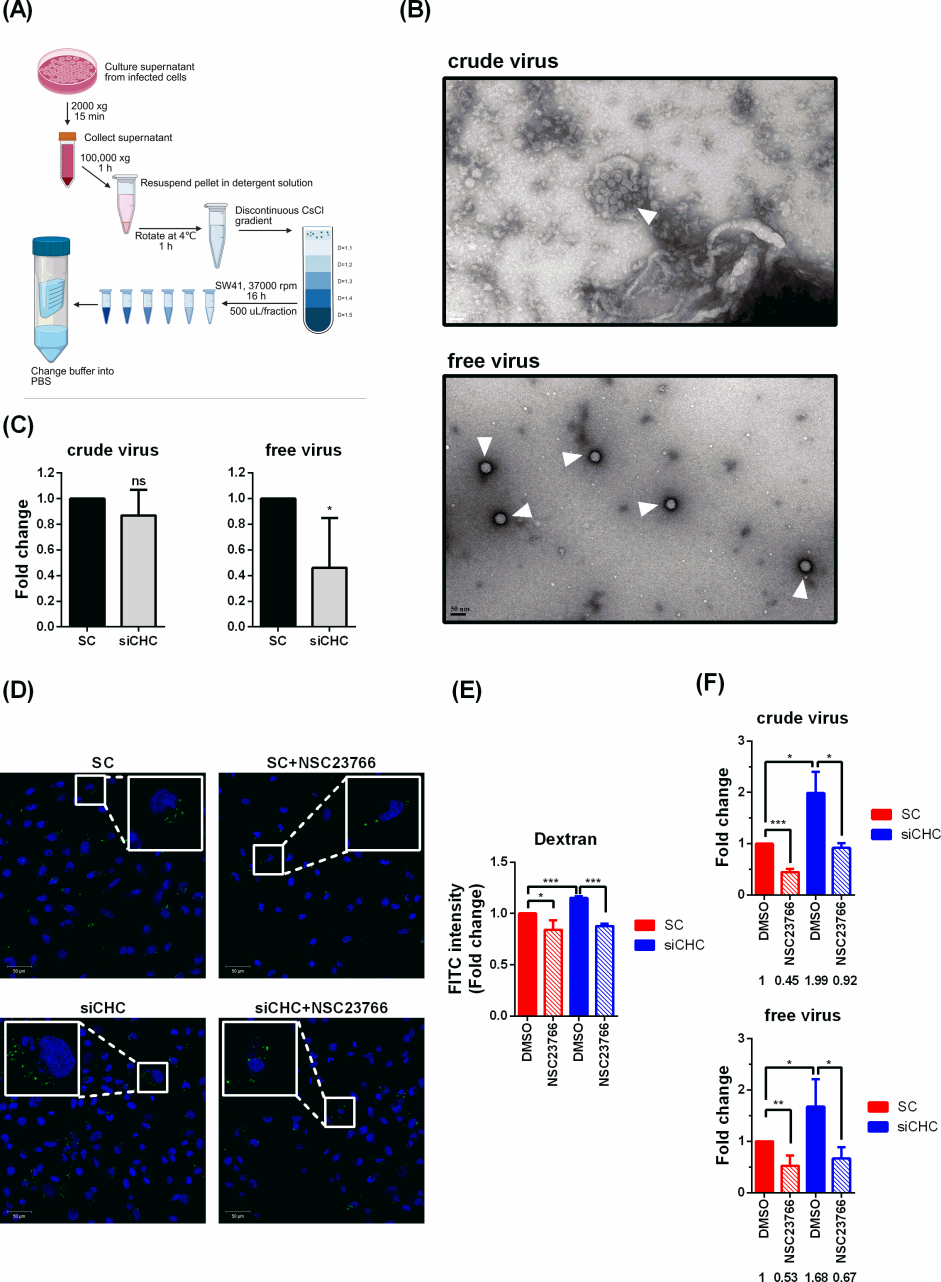
Impact of virus-associated compartments and compensatory macropinocytosis on clathrin-mediated viral entry. (**A**) Schematics for purification of EV-D68 virus particles from the cultured supernatant with detergent and CsCl gradient ultracentrifugation. EV-D68 VP1 expression in each fraction was examined by immunoblotting to verify the distribution of viral particles across the fractions. Fractions 9–12 (density = 1.2–1.4 g/mL) were pooled, concentrated using an Amicon Ultra centrifugal filter, and examined by TEM (**B**). (**C**) siCHC was transfected in RD cells for three days. Crude or CsCl-purified EV-D68 (MOI1) was incubated with cells for 1 h at 4°C. The virus was then removed and the cells were incubated in E2 at 33°C for 1 h. The cells were treated with trypsin prior to harvesting for qRT-PCR analysis. (**D**) Transfected cells were pretreated with NSC23766 for 1 h at 37°C and then incubated with dextran-FITC for 1 h at 33°C. Images were acquired using a confocal microscope. (**E**) Flow cytometric analysis of dextran-FITC uptake in control (SC) and clathrin-depleted (siCHC) cells treated with mock (DMSO) or NSC23766. Data are presented as means ± SD of three replicates. (**F**) Transfected cells were pretreated with 12.5 µM NSC23766 for 2 h at 37°C and inoculated with crude or CsCl-purified (free) EV-D68 (MOI1) for 1 h at 33°C. After removal of virus and inhibitor, cells were incubated for 6 h at 33°C, and viral RNA levels were quantified by qRT-PCR. Data are presented as mean ± SD of three replicates, with mean values indicated. Statistical significance was determined using the Student’s t-test or the Mann–Whitney *U*-test (*, *P* < 0.05; **, *P* < 0.01; and ***, *P* < 0.001).

Based on our findings, both virus particle-associated membrane compartments and compensatory macropinocytosis induced by CME dysfunction may influence EV-D68 entry strategies. To assess the contribution of these factors individually, we separated membrane-associated components from EV-D68 particles. Purification was achieved using detergent treatment (sodium N-lauroyl-sarcosinate) followed by CsCl density gradient centrifugation. The unpurified (crude) virus fraction represents virus obtained using standard enterovirus purification methods, whereas the purified (free) virus fraction underwent multiple purification steps, as outlined in Fig. 7A. Examination by TEM with negative staining revealed that crude virus contained clusters of viral particles and membranous components, consistent with membrane compartments observed at the cell surface in Fig. 5B (upper panel, Fig. 7B). In contrast, CsCl-purified virus displayed distinct, individual virions free of membrane association, making them suitable for downstream experiments (lower panel, Fig. 7B). Comparison of these two virus populations in clathrin-depleted cells revealed differential sensitivity at early infection stages, suggesting that virus-associated membrane compartments can alter entry pathway selection (Fig. 7C).

To further dissect the role of macropinocytosis, we treated cells with NSC23766, a specific Rac1 inhibitor, to suppress Rac1-mediated macropinocytosis. Fluorescence imaging and flow cytometry confirmed that clathrin depletion increased dextran–FITC uptake, whereas NSC23766 treatment reduced it (Fig. 7D and E). Based on the differential sensitivity to clathrin depletion, we further examined whether blockade of CME (via clathrin depletion) and macropinocytosis (via Rac1 inhibition) could effectively restrict infection by crude and CsCl-purified EV-D68. However, although CsCl-purified EV-D68 exhibited sensitivity to clathrin depletion at the entry stage, its infection was not reduced in clathrin-depleted cells. In contrast, NSC23766 treatment significantly reduced infection by both virus preparations in both control and clathrin-depleted cells, indicating that the effects of increased Rac1 activity at the post-entry stage outweighed its contribution during the entry stage (Fig. 7F). Thus, although viral entry was reduced, enhanced Rac1-dependent post-entry efficiency offset the impact of entry restriction.

Together, our data indicate that EV-D68 primarily enters host cells via CME through direct interactions with cellular receptors. Membrane compartment-associated viral particles can utilize macropinocytosis as an alternative entry pathway, particularly under conditions of CME disruption. Additionally, Rac1 activity plays an important role in EV-D68 infection, although further investigation is required to fully define its contribution.

## DISCUSSION

**Fig 8:**
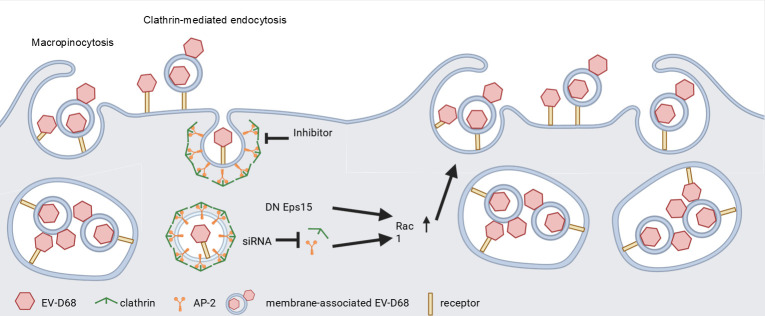
Proposed model illustrating how EV-D68 exploits both CME and macropinocytosis for cellular entry. Individual EV-D68 particles enter cells via CME following receptor attachment, whereas membrane-associated EV-D68 fails to utilize this pathway. Short-term inhibition of CME using chemical inhibitors does not trigger compensatory cellular responses; however, prolonged inhibition—achieved by overexpression of dominant-negative Eps15 or siRNA-mediated knockdown—leads to enhanced macropinocytosis, thereby facilitating EV-D68 entry.

Our findings in this study reveal the complexity of EV-D68 entry, which is influenced not only by receptor but also by the released forms of EV-D68. We found that single EV-D68 particles primarily exploit CME for cell entry, whereas membrane compartment-associated particles preferentially enter through macropinocytosis (illustration in Fig. 8). In previous studies, EV-D68 infection has been understood to begin with viral attachment, followed by receptor-mediated endocytosis and subsequent uncoating in a low-pH environment, which facilitates the release of the viral genome for replication (41). The expression of receptors on the host cell surface is a critical initial determinant of viral entry (14, 20–24, 42). In addition to serving as attachment sites, these receptors often initiate specific endocytic pathways upon binding. For example, the EV-A71 receptors SCARB2 and PSGL1 trigger clathrin-mediated endocytosis and caveolae-dependent endocytosis, respectively, highlighting the role of receptor signaling in guiding the route of viral internalization (25, 26). The EV-D68 strain used in this study demonstrates a strong dependency on sialic acid, suggesting the potential involvement of multiple entry receptors. Studies have demonstrated that EV-D68 strains relying on sialic acid utilize CME for cellular entry, as evidenced by the inhibitory effects of endocytic blockers. Nonetheless, the mechanistic link between receptor binding and internalization remains insufficiently characterized.

Consistent with prior findings on EV-D68 (21, 30), we demonstrated that several chemical inhibitors targeting multiple endocytic pathways exhibited inhibitory effects on EV-D68 infection. Targeting various steps of CME notably inhibited EV-D68 infection, highlighting its role as a dominant pathway (Fig. 1). However, the data also hinted at the involvement of alternative routes, suggesting that multiple entry pathways contribute to the infection of sialic acid-dependent EV-D68 strains. Studies on enterovirus A71, a member of the same family as EV-D68, suggest the possibility of multiple entry pathways (25, 26, 34). The presence of more than one receptor could potentially facilitate diverse entry routes. Additionally, research on IAV, which similarly relies on the same glycans for attachment, demonstrates its ability to utilize at least two endocytic pathways for infection, including both clathrin-mediated endocytosis and macropinocytosis (28, 43–45). Glycans, being modifications on proteins or lipids, play pivotal roles in viral attachment and may also influence uncoating. EV-D68 may engage receptors linked to different endocytic pathways, yet these receptors share similar glycan exposure. This multivalent interaction allows EV-D68 to exploit a variety of host cell types for infection, such as neurons (20, 22, 46). In our investigation of the early-stage dynamics of EV-D68 entry, CME emerged as the dominant pathway, as demonstrated by chemical inhibitor treatments and fluorescence microscopy analysis. Our finding required validation through targeted disruption of key components involved in CME to eliminate the potential non-specific effects of chemical inhibitors. Unexpectedly, our siCHC results showed opposite effects, suggesting additional complexities in the entry mechanisms of EV-D68. To rule out the off-target effects of siRNA, we employed three different siRNA sequences, both individually and in combination, as well as targeting other key proteins involved in CME, including siRNA targeting AP-2 and dominant-negative Eps15 (Fig. 3 and 4). The introduction of siCHC into RD cells resulted in a significant inhibition of transferrin uptake as expected. Nevertheless, none of these approaches produced significantly different outcomes, suggesting that modulation of CME components may exert additional, as yet unidentified effects on EV-D68 infection, or that EV-D68 can utilize multiple entry routes.

TEM images revealed that EV-D68 particles frequently appeared in clusters and were associated with large membrane curvatures that did not resemble classical endocytic structures (Fig. 5). Some studies suggest that certain clathrin-independent endocytosis mechanisms may be triggered under high stimulation (47). It is possible that our sample preparation—designed to enhance viral visibility for TEM—may have inadvertently overstimulated host cells, leading to the induction of atypical membrane dynamics. Additionally, TEM imaging also showed membrane chunks associated with virus particles, highlighting a potential interaction between EV-D68 and membrane compartments. This association is particularly intriguing, as recent studies have demonstrated that even traditionally lytic viruses can exploit non-lytic release and spread mechanisms (35, 48–50). The interplay between virus particles and membranous structures may therefore facilitate alternative modes of viral egress and entry, contributing to the complexity and adaptability of EV-D68 infection. Interestingly, the internalization pathways of enteroviruses are diverse, likely reflecting their heterogeneity in size, surface protein composition, and the characteristics of recipient cells (51–55). This raises the possibility that membrane compartment-associated EV-D68 may exploit phagocytosis as an alternative route of entry into host cells.

Both the complexity of released virus forms and the unidentified compensatory cellular responses may influence virus entry strategies. Long-term inhibition of CME is suspected to induce compensatory changes in other endocytic pathways. Although previous studies have not explicitly examined such mechanisms, indirect evidence indicates that cargo or receptors normally internalized via specific routes can still enter cells through alternative pathways (56–60). Given that CME is a primary mechanism for cellular uptake, surface protein regulation, and plasma membrane maintenance, prolonged CME blockade—such as during a 72-h knockdown—may modulate other endocytic routes. It is therefore important to determine whether the potential alternative entry route for EV-D68 is affected under these conditions. For example, IAV can exploit macropinocytosis under serum-stimulated conditions when dynamin-dependent pathways, including CME, are disrupted, albeit with reduced efficiency (28). Our findings suggest that clathrin knockdown not only disrupts CME but also significantly increases macropinocytosis in both skeletal muscle (RD) and lung carcinoma (A549) cells, reflecting compensatory regulation to maintain cellular homeostasis (Fig. 6). In clathrin-depleted cells, single EV-D68 particles fail to internalize and are presumed to remain adhered to the cell surface (Fig. 7). This raises an important question: are alternative endocytic pathways actively induced in response to these surface-bound virions, or are they triggered by incidental, non-specific mechanisms? Treatment with the Rac1 inhibitor reduced infection by purified EV-D68, which preferentially utilizes CME, suggesting that Rac1 contributes not only to viral entry but may also play additional roles during EV-D68 infection.

Overall, the sialic acid-dependent EV-D68 TW-02795-2014 strain displays heterogeneous populations upon release from host cells, with virus-membrane associations potentially influencing subsequent attachment and entry. The presence of diverse EV-D68 release forms, along with compensatory crosstalk between endocytic pathways, contributes to the complexity of viral entry. These findings highlight the importance of endocytic plasticity in viral infection and underscore the need to consider compensatory mechanisms when investigating viral entry strategies. It also suggests that effective antiviral strategies against EV-D68 should not only target receptor binding but also account for the heterogeneous nature of virus release and the flexibility of endocytic entry routes.

## MATERIALS AND METHODS

### Cell culture

Human embryonal rhabdomyosarcoma (RD) cells and human lung carcinoma A549 cells were maintained in Dulbecco’s modified Eagle medium (DMEM; Thermo Fisher Scientific, CA, USA) comprising 10% fetal bovine serum (FBS; Sigma-Aldrich, St Louis, MO, USA), 0.1 mg/mL streptomycin, and 100 U/mL penicillin.

### Antibodies

Rabbit anti-EVD68 VP1 antibody (Cat. GTX132313), rabbit anti-α-tubulin antibody (GTX112141), rabbit anti-rac1 antibody (GTX100761), goat anti-mouse IgG antibody (HRP) (Cat. GTX213111-01), Easyblot anti-rabbit IgG (HRP) (Cat. GTX221666-01) used in immunoblotting were purchased from GeneTex (Irvine, CA, USA). Rabbit anti-GFP antibody (Cat. Sc-8334) used in immunoblotting was purchased from Santa Cruz (Santa Cruz, CA, USA). goat anti-mouse IgG (H + L) secondary antibody, Alexa Fluor 488 conjugate (Cat. A11001), donkey anti-rabbit IgG (H + L) secondary antibody, Alexa Fluor 568 (Cat. A10042) used in the immunofluorescence assay were purchased from Thermo Fisher Scientific. Anti-clathrin heavy chain (CHC) antibody TD.1 (immunoblotting) and X22 (immunofluorescence) were generated from mouse ascites (61, 62).

### Virus amplification

EV-D68 isolate TW-02795-2014 (GenBank: KT711088) was amplified in RD cells. RD cells were infected with EV-D68 at an MOI of 0.1 in DMEM and incubated for 1 h at 33°C. Subsequently, the virus-containing media were aspirated, and the cells were washed with phosphate-buffered saline (PBS). The cells were then cultured in DMEM supplemented with 2% FBS (E2) and incubated at 33°C until a cytopathic effect in approximately 90% of the cells.

### Virus purification

For general freeze-and-thaw approaches, after observing a cytopathic effect in over 90% of the cells, adherent cells were scratched with a rubber policeman, and all the cells and the culture media were collected. The collected material was then centrifuged at 300 × *g* at 4 for 10 min. The supernatant was transferred to a new tube. Subsequently, the pellet was frozen with liquid nitrogen and thawed in a water bath at 37, and this freeze-thaw cycle was repeated three times. The supernatant and thawed pellet were mixed and centrifuged at 300 × *g* at 4 for 10 min. The resulting supernatant, designated as crude virus, was aliquoted and stored at −80°C for subsequent experiments. For TEM analysis, the virus was concentrated and buffer-exchanged into PBS using a 100 kDa molecular weight cutoff (MWCO) Amicon Ultra centrifugal filter (Cat. UFC910096, Millipore, Darmstadt, Germany).

For the free virus (single virus) purification by CsCl density gradient approach (illustration in Fig. 7A), the culture medium from EV-D68-infected cells was collected and centrifuged at 2,000 × *g* for 15 min at 4°C to remove dead cells and debris. The supernatant was then centrifuged at 100,000 × *g* for 1 h at 4°C to pellet all the viruses. Discard the supernatant and gently resuspend the pellet in a detergent-containing buffer composed of 5 mM MgCl_2_, 10 μg/mL DNase, 7.5 mg/mL RNase, 0.8 mg/mL trypsin, 15 mM EDTA (pH 9.5), and 1% (wt/vol) sodium N-lauroyl-sarcosinate in buffer A (250 mM NaCl, 250 mM HEPES, pH 7.5) (63). The mixture was incubated at 4°C on an overhead rotator for 1 h. Concurrently, five different densities of CsCl solutions were prepared in the PBS buffer. The solutions of 2 mL each were layered to create the density gradient from 1.1 to 1.5 using a frozen approach and peristaltic pump in a 9/16 × 3½ PP tube. Subsequently, 1 mL of the virus solution was carefully loaded onto the top of the CsCl density gradient. The gradient was centrifuged at 37,000 rpm for 16 h at 4°C using an SW41 Ti rotor. Following centrifugation, 500 μL fractions were collected sequentially from the top. The target fraction was concentrated and buffer-exchanged into PBS using a 100 kDa MWCO Amicon Ultra filter.

### Virus titer determination

RD cells were infected with five serial dilutions of viruses in DMEM and incubated at 33°C for 1 h. Following incubation, the viral inoculum was removed, and the cells were overlaid with DMEM containing 0.3% agarose. After a 48-h incubation at 33°C, the cells were fixed with 4% paraformaldehyde (PFA) and stained with 1% crystal violet to visualize plaques.

### Immunoblotting analysis

Equal amounts of proteins extracted from RD and A549 cell lysates were separated by SDS-PAGE and transferred onto polyvinylidene fluoride membranes. Specific proteins were detected using the primary and secondary antibodies described previously. Chemiluminescent signals were visualized using either autoradiography film or a FluorChem HD system (ProteinSimple, San Jose, CA, USA). Band intensities were quantified using ImageJ or AlphaView SA software to assess protein expression levels.

### Viral RNA measurement

The cells were harvested, and RNA was extracted using TRIzol reagent (Thermo Fisher Scientific) following the manufacturer’s instructions. Subsequently, the extracted RNA was reverse transcribed using random hexamers and M-MLV reverse transcriptase (Thermo Fisher Scientific) according to the manufacturer’s instructions to generate complementary DNA. For measuring the expression levels of the genes of interest, specific primers and Smart Quant Green Master Mix (Protech, Taipei, Taiwan) were used in a quantitative real-time polymerase chain reaction (qRT-PCR) assay. The primer sequences were listed in Table S1. All the expression levels of the genes of interest were normalized to the expression level of GAPDH in the relative quantification results.

### Neuraminidase inhibition

RD cells were cultured in DMEM supplemented with the neuraminidase (Cat. N2876, Sigma) at a concentration of 50 mU per 5 × 10^5^ cells at 37 for 1 h. The cells were infected with the virus in DMEM at an MOI of 10 and incubated at 33°C for 1 h. The medium was then replaced with E2, followed by a 6-h incubation at 33°C before harvesting for immunoblot analysis.

### SNA competition

RD cells were pre-cooled at 4 for 10 min and then pre-treated with 25 μg/mL SNA (Cat. FL-1301, Vector Laboratories, Burlingame, CA, USA) or MAL-I (Cat. FL-1311, Vector Laboratories) at 4°C for 30 min. The cells were infected with EV-D68 at a MOI of 1 at 4°C for 30 min. Samples were either harvested immediately or incubated in E2 at 33°C for 6 h before collection.

### Chemical inhibitors treatment

Various inhibitors were acquired from Sigma or MCE (NJ, USA), with their working concentration determined based on previous studies. The inhibitors used included 20 μM CPZ (Cat. C8138, Sigma), 40 μg/mL nystatin (Cat. N4014, Sigma), 40 μM genistein (Cat. G669, Sigma), 25 μM EIPA (Cat. A3085, Sigma), 4 μM CB (Cat. C6762, Sigma), 80 μM Dynasore (Cat. SML0340, Sigma), 200 nM BafA1 (Cat. B1793, Sigma), 40 nM CQ (Cat. C6628, Sigma), 50 μM NH_4_Cl (Cat. A5666, Sigma), and 12.5 μM NSC23766 (Cat. MCE-HY-15723, MCE). RD cells and A549 cells were cultured in DMEM supplemented with the aforementioned inhibitors at 37°C for 2 h. Following this incubation, the culture media were removed. The EV-D68 was diluted in DMEM containing the abovementioned inhibitors at an MOI of 1, 40, or 100 and added to the cells, which were then incubated at 33°C for 1 h. After incubation with the virus, the medium was replaced with E2. The cells were either further incubated at 33°C for 6 h or harvested immediately, followed by analysis using immunoblotting, qRT-PCR, or plaque assay.

### Immunofluorescence assay

In general, RD cells were infected with EV-D68 at an MOI of 200 at 33°C for 1 h. After infection, the cells were washed with PBS to remove unbound virus and then fixed with 4% PFA. For synchronization of entry events, the cells were pre-cooled at 4°C for 10 min and infected with EV-D68 at an MOI of 100 for 15 min at 4°C. The cells were then shifted to 33°C and incubated for 45 min. In the chemical inhibitor treatment groups, the inhibitors were added after the 15-min adsorption period at 4°C and co-incubated with the virus during the subsequent 33°C incubation. After viral infection, the cells were fixed with 4% PFA, permeabilized with 0.5% Triton X-100, and subjected to antibody staining. Imaging was performed using a Zeiss LSM 780 laser scanning confocal microscope. Fluorescence intensity and co-localization efficiency were quantified using Zen software to determine the total signal within individual cells.

### RNA interference

siRNAs targeting CHC, AP2M1, and Rac1 were obtained from Invitrogen and OmicsBio (New Taipei City, Taiwan), with sequences listed in Table S2. RD and A549 cells were transfected with siRNAs using Lipofectamine RNAiMAX (Cat. 13778075, Thermo Fisher Scientific) following the manufacturer’s instructions, and experiments were conducted 3 days post-transfection. For comparisons between crude and free virus infections, transfected cells were pre-cooled at 4°C for 10 min, then infected with the respective viruses at 4°C for 1 h. After removing unbound viruses, the cells were incubated in DMEM at 33°C for 1 h. The cells were trypsinized at 33°C, diluted with PBS, collected by centrifugation at 300 × *g* for 10 min, and the resulting pellets were processed with TRIzol reagent for RNA extraction.

### Dominant-negative mutant Eps15 overexpression

RD cells were transfected with the EGFP-C2 or mutant Eps15 plasmid using X-tremeGENE (Cat. XTGHP-RO, Sigma) (33). Sixteen hours post-transfection, the cells were infected with EV-D68 at an MOI of 1 for 1 h at 33°C. After infection, the cells were incubated in E2 at 33°C for 6 h. Cell lysates were then collected for immunoblotting analysis.

### Transferrin and dextran uptake

RD cells were co-treated with Hoechst dye and either 5 μg/mL of transferrin-488 (Cat. T13342, Invitrogen) or 1 mg/mL of dextran-488 (Cat. 46945, Sigma) at 33°C for 1 h. After the incubation, the cells were washed and mounted onto slides for imaging. Fluorescent signals were observed and captured using a Zeiss LSM 780. The acquired images were then quantified using Investigator software, allowing for the analysis and quantification of fluorescence puncta numbers within the cells. For flow cytometric analysis of dextran-FITC uptake, the cells were washed with PBS and trypsinized after 1 h of incubation. The cells were collected by centrifugation at 500 × *g* and resuspended in PBS. Data acquisition was performed using an Attune NxT Flow Cytometer (Thermo Fisher Scientific), and dextran-FITC fluorescence intensity was quantified from 10,000 gated events per sample.

### Transmission electron microscopy and sample preparation

For negative staining, samples were loaded onto carbon film-coated copper grids (Cat. TEM-CF200CU50, Sigma) and stained with 2% uranyl acetate. For embedded samples preparation, RD cells were seeded onto 7.8 mil embedding film and infected with virus at an MOI of 7,000 for 1 h at 33°C. Following infection, the cells were fixed with 2.5% glutaraldehyde in 0.15 M cacodylate buffer. Post-fixation and staining were performed using 1% osmium tetroxide and 1.5% potassium ferrocyanide in 0.3 M cacodylate buffer containing 4 mM calcium chloride. The cells were then incubated with Walton’s lead aspartate solution, followed by a graded ethanol dehydration series. After dehydration, samples were infiltrated and embedded in Spurr’s resin. Ultrathin sections were prepared using a Leica EM UC7 ultramicrotome and collected on 200-mesh copper grids. Negative staining samples and sections were examined using a JEOL JEM-1230 transmission electron microscope.

### Statistical analysis

All experiments were conducted independently at least three times. Data are presented as the mean ± standard deviation (SD). For immunofluorescence analysis, more than 100 cells were counted per group. Statistical analyses were performed using unpaired Student’s *t*-test and Mann–Whitney *U*-test in GraphPad Prism 6.01. Statistical significance is indicated as follows: *P* < 0.05 (*), *P* < 0.01 (**), and *P* < 0.001 (***), no significance (ns).

## Data Availability

The complete genomic sequence of the EV-D68 isolate used in this study is available in the NCBI database under accession number KT711088.1. The plasmid Eps15 (Δ95–295)/GFP-C2 was constructed by Alice Dautry-Varsat and previously described in Benmerah et al. (33). Gene information for CHC, AP2M1, and Rac1 targeted by siRNA can be found in the NCBI database under Gene IDs 1213, 1173, and 5879, respectively. All other data supporting the findings of this study are available within the article.

## SUPPLEMENTAL MATERIAL

Tables S1 and S2 jvi.00358-26-s0001.docx Primer sequences and siRNA sequences.

ASM does not own the copyrights to Supplemental Material that may be linked to, or accessed through, an article. The authors have granted ASM a non-exclusive, world-wide license to publish the Supplemental Material files. Please contact the corresponding author directly for reuse.

## References

[1] Zell R, Delwart E, Gorbalenya AE, Hovi T, King AMQ, Knowles NJ, Lindberg AM, Pallansch MA, Palmenberg AC, Reuter G, Simmonds P, Skern T, Stanway G, Yamashita T. 2017. ICTV virus taxonomy profile: picornaviridae. J Gen Virol 98:2421–2422. doi:10.1099/jgv.0.000911 28884666 PMC5725991

[2] Ishiko H, Miura R, Shimada Y, Hayashi A, Nakajima H, Yamazaki S, Takeda N. 2002. Human rhinovirus 87 identified as human enterovirus 68 by VP4-based molecular diagnosis. Intervirology 45:136–141. doi:10.1159/000065866 12403917

[3] Schieble JH, Fox VL, Lennette EH. 1967. A probable new human picornavirus associated with respiratory diseases. Am J Epidemiol 85:297–310. doi:10.1093/oxfordjournals.aje.a120693 4960233

[4] Piralla A, Girello A, Grignani M, Gozalo-Margüello M, Marchi A, Marseglia G, Baldanti F. 2014. Phylogenetic characterization of enterovirus 68 strains in patients with respiratory syndromes in Italy. J Med Virol 86:1590–1593. doi:10.1002/jmv.23821 24155220 PMC7166609

[5] Lu QB, Wo Y, Wang HY, Wei MT, Zhang L, Yang H, Liu EM, Li TY, Zhao ZT, Liu W, Cao WC. 2014. Detection of enterovirus 68 as one of the commonest types of enterovirus found in patients with acute respiratory tract infection in China. J Med Microbiol 63:408–414. doi:10.1099/jmm.0.068247-0 24324030

[6] Reiche J, Böttcher S, Diedrich S, Buchholz U, Buda S, Haas W, Schweiger B, Wolff T. 2015. Low-level circulation of enterovirus D68-associated acute respiratory infections, Germany, 2014. Emerg Infect Dis 21:837–841. doi:10.3201/eid2105.141900 25898320 PMC4412236

[7] Messacar K, Schreiner TL, Maloney JA, Wallace A, Ludke J, Oberste MS, Nix WA, Robinson CC, Glodé MP, Abzug MJ, Dominguez SR. 2015. A cluster of acute flaccid paralysis and cranial nerve dysfunction temporally associated with an outbreak of enterovirus D68 in children in Colorado, USA. Lancet 385:1662–1671. doi:10.1016/S0140-6736(14)62457-0 25638662

[8] Park SW, Pons-Salort M, Messacar K, Cook C, Meyers L, Farrar J, Grenfell BT. 2021. Epidemiological dynamics of enterovirus D68 in the United States and implications for acute flaccid myelitis. Sci Transl Med 13:eabd2400. doi:10.1126/scitranslmed.abd2400 33692131

[9] Sahay G, Alakhova DY, Kabanov AV. 2010. Endocytosis of nanomedicines. J Control Release 145:182–195. doi:10.1016/j.jconrel.2010.01.036 20226220 PMC2902597

[10] Edeling MA, Smith C, Owen D. 2006. Life of a clathrin coat: insights from clathrin and AP structures. Nat Rev Mol Cell Biol 7:32–44. doi:10.1038/nrm1786 16493411

[11] Kirchhausen T. 2009. Imaging endocytic clathrin structures in living cells. Trends Cell Biol 19:596–605. doi:10.1016/j.tcb.2009.09.002 19836955 PMC2796618

[12] Ehrlich M, Boll W, Van Oijen A, Hariharan R, Chandran K, Nibert ML, Kirchhausen T. 2004. Endocytosis by random initiation and stabilization of clathrin-coated pits. Cell 118:591–605. doi:10.1016/j.cell.2004.08.017 15339664

[13] Ferguson SM, De Camilli P. 2012. Dynamin, a membrane-remodelling GTPase. Nat Rev Mol Cell Biol 13:75–88. doi:10.1038/nrm3266 22233676 PMC3519936

[14] Lamaze C, Tardif N, Dewulf M, Vassilopoulos S, Blouin CM. 2017. The caveolae dress code: structure and signaling. Curr Opin Cell Biol 47:117–125. doi:10.1016/j.ceb.2017.02.014 28641181

[15] Sandvig K, Torgersen ML, Raa HA, van Deurs B. 2008. Clathrin-independent endocytosis: from nonexisting to an extreme degree of complexity. Histochem Cell Biol 129:267–276. doi:10.1007/s00418-007-0376-5 18193449 PMC2248609

[16] Ferreira APA, Boucrot E. 2018. Mechanisms of carrier formation during clathrin-independent endocytosis. Trends Cell Biol 28:188–200. doi:10.1016/j.tcb.2017.11.004 29241687

[17] Doherty GJ, McMahon HT. 2009. Mechanisms of endocytosis. Annu Rev Biochem 78:857–902. doi:10.1146/annurev.biochem.78.081307.110540 19317650

[18] Chu VC, Whittaker GR. 2004. Influenza virus entry and infection require host cell N-linked glycoprotein. Proc Natl Acad Sci USA 101:18153–18158. doi:10.1073/pnas.0405172102 15601777 PMC535801

[19] Imamura T, Okamoto M, Nakakita S, Suzuki A, Saito M, Tamaki R, Lupisan S, Roy CN, Hiramatsu H, Sugawara K, Mizuta K, Matsuzaki Y, Suzuki Y, Oshitani H. 2014. Antigenic and receptor binding properties of enterovirus 68. J Virol 88:2374–2384. doi:10.1128/JVI.03070-13 24371050 PMC3958110

[20] Pereirinha da Silva AK, van Trijp JP, Montenarie A, Fok JA, Sooksawasdi Na Ayudhya S, Pieters RJ, Boons G-J, van Riel D, de Vries RP, Bauer L. 2025. Sialic acid-containing glycolipids extend the receptor repertoire of enterovirus-D68. ACS Infect Dis 11:2090–2103. doi:10.1021/acsinfecdis.5c00063 40633911 PMC12340969

[21] Baggen J, Liu Y, Lyoo H, van Vliet ALW, Wahedi M, de Bruin JW, Roberts RW, Overduin P, Meijer A, Rossmann MG, Thibaut HJ, van Kuppeveld FJM. 2019. Bypassing pan-enterovirus host factor PLA2G16. Nat Commun 10:3171. doi:10.1038/s41467-019-11256-z 31320648 PMC6639302

[22] Wei W, Guo H, Chang J, Yu Y, Liu G, Zhang N, Willard SH, Zheng S, Yu XF. 2016. ICAM-5/telencephalin is a functional entry receptor for enterovirus D68. Cell Host Microbe 20:631–641. doi:10.1016/j.chom.2016.09.013 27923705

[23] Varanese L, Xu L, Peters CE, Pintilie G, Roberts DS, Raj S, Liu M, Ooi YS, Diep J, Qiao W, Richards CM, Callaway J, Bertozzi CR, Jabs S, de Vries E, van Kuppeveld FJM, Nagamine CM, Chiu W, Carette JE. 2025. MFSD6 is an entry receptor for enterovirus D68. Nature 641:1268–1275. doi:10.1038/s41586-025-08908-0 40132641 PMC12333957

[24] Liu X, Li H, Li Z, Gao D, Zhou J, Ni F, Yu Q, Huang Y, Tang Y, Xue L, Wang S, Yang J, Guo H, Wang Y, Yu XF, Yu Z, Wei W. 2025. MFSD6 is an entry receptor for respiratory enterovirus D68. Cell Host Microbe 33:267–278. doi:10.1016/j.chom.2024.12.015 39798568

[25] Lin YW, Lin HY, Tsou YL, Chitra E, Hsiao KN, Shao HY, Liu CC, Sia C, Chong P, Chow YH. 2012. Human SCARB2-mediated entry and endocytosis of EV71. PLoS One 7:e30507. doi:10.1371/journal.pone.0030507 22272359 PMC3260287

[26] Lin HY, Yang YT, Yu SL, Hsiao KN, Liu CC, Sia C, Chow YH. 2013. Caveolar endocytosis is required for human PSGL-1-mediated enterovirus 71 infection. J Virol 87:9064–9076. doi:10.1128/JVI.00573-13 23760234 PMC3754029

[27] Sieben C, Sezgin E, Eggeling C, Manley S. 2020. Influenza A viruses use multivalent sialic acid clusters for cell binding and receptor activation. PLoS Pathog 16:e1008656. doi:10.1371/journal.ppat.1008656 32639985 PMC7371231

[28] de Vries E, Tscherne DM, Wienholts MJ, Cobos-Jiménez V, Scholte F, García-Sastre A, Rottier PJM, de Haan CAM. 2011. Dissection of the influenza A virus endocytic routes reveals macropinocytosis as an alternative entry pathway. PLoS Pathog 7:e1001329. doi:10.1371/journal.ppat.1001329 21483486 PMC3068995

[29] Rossman JS, Leser GP, Lamb RA. 2012. Filamentous influenza virus enters cells via macropinocytosis. J Virol 86:10950–10960. doi:10.1128/JVI.05992-11 22875971 PMC3457176

[30] Jiang Y, Liu S, Shen S, Guo H, Huang H, Wei W. 2020. Methyl-β-cyclodextrin inhibits EV-D68 virus entry by perturbing the accumulation of virus particles and ICAM-5 in lipid rafts. Antiviral Res 176:104752. doi:10.1016/j.antiviral.2020.104752 32101770

[31] Gong Y-N, Yang S-L, Shih S-R, Huang Y-C, Chang P-Y, Huang C-G, Kao K-C, Hu H-C, Liu Y-C, Tsao K-C. 2016. Molecular evolution and the global reemergence of enterovirus D68 by genome-wide analysis. Medicine (Abingdon) 95:e4416. doi:10.1097/MD.0000000000004416 PMC4979813 27495059

[32] Hussain KM, Leong KLJ, Ng MM-L, Chu JJH. 2011. The essential role of clathrin-mediated endocytosis in the infectious entry of human enterovirus 71. J Biol Chem 286:309–321. doi:10.1074/jbc.M110.168468 20956521 PMC3012988

[33] Benmerah A, Bayrou M, Cerf-Bensussan N, Dautry-Varsat A. 1999. Inhibition of clathrin-coated pit assembly by an Eps15 mutant. J Cell Sci 112 (Pt 9):1303–1311. doi:10.1242/jcs.112.9.1303 10194409

[34] Chen SL, Liu YG, Zhou YT, Zhao P, Ren H, Xiao M, Zhu YZ, Qi ZT. 2019. Endophilin-A2-mediated endocytic pathway is critical for enterovirus 71 entry into caco-2 cells. Emerg Microbes Infect 8:773–786. doi:10.1080/22221751.2019.1618686 31132962 PMC6542187

[35] Rudy MJ, Coughlan C, Hixon AM, Clarke P, Tyler KL. 2022. Density analysis of enterovirus D68 shows viral particles can associate with exosomes. Microbiol Spectr 10:e0245221. doi:10.1128/spectrum.02452-21 35170992 PMC8849102

[36] Blanco JCG, Sylla FYD, Granados S, Noghero A, Boukhvalova MS, Kajon AE. 2025. Enterovirus D68 infection in cotton rats results in systemic inflammation with detectable viremia associated with extracellular vesicle and neurologic disease. Sci Rep 15:6514. doi:10.1038/s41598-025-89447-6 39987168 PMC11847025

[37] Fu Y, Xiong S. 2023. Exosomes mediate Coxsackievirus B3 transmission and expand the viral tropism. PLoS Pathog 19:e1011090. doi:10.1371/journal.ppat.1011090 36634130 PMC9888687

[38] Gao P, Zhou L, Wu J, Weng W, Wang H, Ye M, Qu Y, Hao Y, Zhang Y, Ge X, Guo X, Han J, Yang H. 2023. Riding apoptotic bodies for cell-cell transmission by African swine fever virus. Proc Natl Acad Sci USA 120:e2309506120. doi:10.1073/pnas.2309506120 37983498 PMC10691326

[39] Kuriyama M, Hirose H, Masuda T, Shudou M, Arafiles JVV, Imanishi M, Maekawa M, Hara Y, Futaki S. 2022. Piezo1 activation using Yoda1 inhibits macropinocytosis in A431 human epidermoid carcinoma cells. Sci Rep 12:6322. doi:10.1038/s41598-022-10153-8 35428847 PMC9012786

[40] Gao Y, Dickerson JB, Guo F, Zheng J, Zheng Y. 2004. Rational design and characterization of a Rac GTPase-specific small molecule inhibitor. Proc Natl Acad Sci USA 101:7618–7623. doi:10.1073/pnas.0307512101 15128949 PMC419655

[41] Elrick MJ, Pekosz A, Duggal P. 2021. Enterovirus D68 molecular and cellular biology and pathogenesis. J Biol Chem 296:100317. doi:10.1016/j.jbc.2021.100317 33484714 PMC7949111

[42] Liu Y, Sheng J, Baggen J, Meng G, Xiao C, Thibaut HJ, van Kuppeveld FJM, Rossmann MG. 2015. Sialic acid-dependent cell entry of human enterovirus D68. Nat Commun 6:8865. doi:10.1038/ncomms9865 26563423 PMC4660200

[43] Eierhoff T, Hrincius ER, Rescher U, Ludwig S, Ehrhardt C. 2010. The epidermal growth factor receptor (EGFR) promotes uptake of influenza A viruses (IAV) into host cells. PLoS Pathog 6:e1001099. doi:10.1371/journal.ppat.1001099 20844577 PMC2936548

[44] Vieira AV, Lamaze C, Schmid SL. 1996. Control of EGF receptor signaling by clathrin-mediated endocytosis. Science 274:2086–2089. doi:10.1126/science.274.5295.2086 8953040

[45] Rust MJ, Lakadamyali M, Zhang F, Zhuang X. 2004. Assembly of endocytic machinery around individual influenza viruses during viral entry. Nat Struct Mol Biol 11:567–573. doi:10.1038/nsmb769 15122347 PMC2748740

[46] Rosenfeld AB, Warren AL, Racaniello VR. 2019. Neurotropism of enterovirus D68 isolates is independent of sialic acid and is not a recently acquired phenotype. mBio 10. doi:10.1128/mBio.02370-19 PMC6805996 31641090

[47] Mayor S, Parton RG, Donaldson JG. 2014. Clathrin-independent pathways of endocytosis. Cold Spring Harb Perspect Biol 6:a016758. doi:10.1101/cshperspect.a016758 24890511 PMC4031960

[48] Bird SW, Maynard ND, Covert MW, Kirkegaard K. 2014. Nonlytic viral spread enhanced by autophagy components. Proc Natl Acad Sci USA 111:13081–13086. doi:10.1073/pnas.1401437111 25157142 PMC4246951

[49] Huang HI, Lin JY, Chiang HC, Huang PN, Lin QD, Shih SR. 2020. Exosomes facilitate transmission of enterovirus A71 from human intestinal epithelial cells. J Infect Dis 222:456–469. doi:10.1093/infdis/jiaa174 32271384 PMC7336570

[50] Taylor MP, Burgon TB, Kirkegaard K, Jackson WT. 2009. Role of microtubules in extracellular release of poliovirus. J Virol 83:6599–6609. doi:10.1128/JVI.01819-08 19369338 PMC2698579

[51] Horibe S, Tanahashi T, Kawauchi S, Murakami Y, Rikitake Y. 2018. Mechanism of recipient cell-dependent differences in exosome uptake. BMC Cancer 18:47. doi:10.1186/s12885-017-3958-1 29306323 PMC5756423

[52] Koumangoye RB, Sakwe AM, Goodwin JS, Patel T, Ochieng J. 2011. Detachment of breast tumor cells induces rapid secretion of exosomes which subsequently mediate cellular adhesion and spreading. PLoS One 6:e24234. doi:10.1371/journal.pone.0024234 21915303 PMC3167827

[53] Feng D, Zhao WL, Ye YY, Bai XC, Liu RQ, Chang LF, Zhou Q, Sui SF. 2010. Cellular internalization of exosomes occurs through phagocytosis. Traffic 11:675–687. doi:10.1111/j.1600-0854.2010.01041.x 20136776

[54] Fitzner D, Schnaars M, van Rossum D, Krishnamoorthy G, Dibaj P, Bakhti M, Regen T, Hanisch U-K, Simons M. 2011. Selective transfer of exosomes from oligodendrocytes to microglia by macropinocytosis. J Cell Sci 124:447–458. doi:10.1242/jcs.074088 21242314

[55] Emam SE, Ando H, Lila ASA, Shimizu T, Okuhira K, Ishima Y, Mahdy MA, Ghazy F-ES, Sagawa I, Ishida T. 2018. Liposome co-incubation with cancer cells secreted exosomes (extracellular vesicles) with different proteins expressions and different uptake pathways. Sci Rep 8:14493. doi:10.1038/s41598-018-32861-w 30262875 PMC6160473

[56] Puri C. 2009. Loss of myosin VI no insert isoform (NoI) induces a defect in clathrin-mediated endocytosis and leads to caveolar endocytosis of transferrin receptor. J Biol Chem 284:34998–35014. doi:10.1074/jbc.M109.012328 19840950 PMC2787362

[57] Orlichenko L, Weller SG, Cao H, Krueger EW, Awoniyi M, Beznoussenko G, Buccione R, McNiven MA. 2009. Caveolae mediate growth factor-induced disassembly of adherens junctions to support tumor cell dissociation. Mol Biol Cell 20:4140–4152. doi:10.1091/mbc.e08-10-1043 19641024 PMC2754928

[58] Di Guglielmo GM, Le Roy C, Goodfellow AF, Wrana JL. 2003. Distinct endocytic pathways regulate TGF-beta receptor signalling and turnover. Nat Cell Biol 5:410–421. doi:10.1038/ncb975 12717440

[59] Hernández-Deviez DJ, Howes MT, Laval SH, Bushby K, Hancock JF, Parton RG. 2008. Caveolin regulates endocytosis of the muscle repair protein, dysferlin. J Biol Chem 283:6476–6488. doi:10.1074/jbc.M708776200 18096699

[60] Utskarpen A, Massol R, van Deurs B, Lauvrak SU, Kirchhausen T, Sandvig K. 2010. Shiga toxin increases formation of clathrin-coated pits through Syk kinase. PLoS One 5:e10944. doi:10.1371/journal.pone.0010944 20668539 PMC2910670

[61] Näthke IS, Heuser J, Lupas A, Stock J, Turck CW, Brodsky FM. 1992. Folding and trimerization of clathrin subunits at the triskelion hub. Cell 68:899–910. doi:10.1016/0092-8674(92)90033-9 1547490

[62] Brodsky FM. 1985. Clathrin structure characterized with monoclonal antibodies. II. Identification of in vivo forms of clathrin. J Cell Biol 101:2055–2062. doi:10.1083/jcb.101.6.2055 4066749 PMC2114020

[63] Liu Y, Sheng J, van Vliet ALW, Buda G, van Kuppeveld FJM, Rossmann MG. 2018. Molecular basis for the acid-initiated uncoating of human enterovirus D68. Proc Natl Acad Sci USA 115. doi:10.1073/pnas.1803347115 PMC6310856 30530701

