# Supplementary material for: EV-D68 exploits clathrin-mediated endocytosis and compensatory macropinocytosis for cellular entry
Source: J Virol. 2026 Apr 27;100(5):e00358-26. doi: 10.1128/jvi.00358-26 (PMC13185571; doi:10.1128/jvi.00358-26)
Supplement: Tables S1 and S2 — Primer sequences and siRNA sequences. [file jvi.00358-26-s0001.docx]

**Table S1 Primer used for qRT-PCR**

| Primer | sequence |
| --- | --- |
| GAPDH-F | 5’-AAG AAG GTG GTG AAG CAG GC-3’ |
| GAPDH-R | 5’-TCC ACC ACC CTG TTG CTG TA-5’ |
| EVD68/2795-VP1-F | 5’-GCA ACT GAC ACC GTG AAA AGT-3’ |
| EVD68/2795-VP1-R | 5’-AGT TTC GGATAC ACC GTG TTG-3’ |
| CHC-F | 5’-GGA AAC TTG GAT CGG GCA TAT GAG T-3’ |
| CHC-R | 5’-CCA TTC CTT TCT GCA ACT GGG C-3’ |
| AP2M1-F | 5’-CTG GCG GCG AGA GGG TAT CA-3’ |
| AP2M1-R | 5’-GCG CGA ATG GCA CCT CAA AG-3’ |

**Table S2** **siRNA sequence**

| siRNA | siRNA sequence |
| --- | --- |
| siCHC-1 | 5’-CCG GAA AUU UGA UGU CAA UAC UUC A-3’ |
| siCHC-2 | 5’-CCG CCU UGC AGA GUU AGA AGA AUU U-3’ |
| siCHC-3 | 5’-GAG UGC UUU GGA GCU UGU CUG UUU A-3’ |
| siAP2M1-1 | 5’-GUG GUC AUC AAG UCC AAC UUU TT-3’ |
| siAP2M1-2 | 5’-CAC CAG CUU CUU CCA CGU UAA TT-3’ |
| siAP2M1-3 | 5’-GCU GGA UGA GAU UCU AGA CUU TT-3’ |
| siRac1-1 | 5’-CCU AUC CUA UCC GCA AAC ATT-3’ |
| siRac1-2 | 5’-GAU AAA GAC ACG AUC GAG ATT-3’ |
| siRac1-3 | 5’-CCU GGA GAA UAU AUC CCU ATT-3’ |
